# Supplementary material for: Predictive Prioritization of Enhancers Associated with Pancreas Disease Risk
Source: bioRxiv. 2024 Sep 13:2024.09.07.611794. Preprint. [Version 1] doi: 10.1101/2024.09.07.611794 (PMC11418953; doi:10.1101/2024.09.07.611794)
Supplement: 1 [file NIHPP2024.09.07.611794V1-supplement-1.pdf]

bioRxiv preprint doi: https://doi.org/10.1101/2024.09.07.611794; this version posted September 13, 2024. The copyright holder for this preprint (which was not certified by peer review) is the author/funder. This article is a US Government work. It is not subject to copyright under 17 USC 105 and is also made available for use under a CC0 license.

Wang et al, Supplementary Figure 1

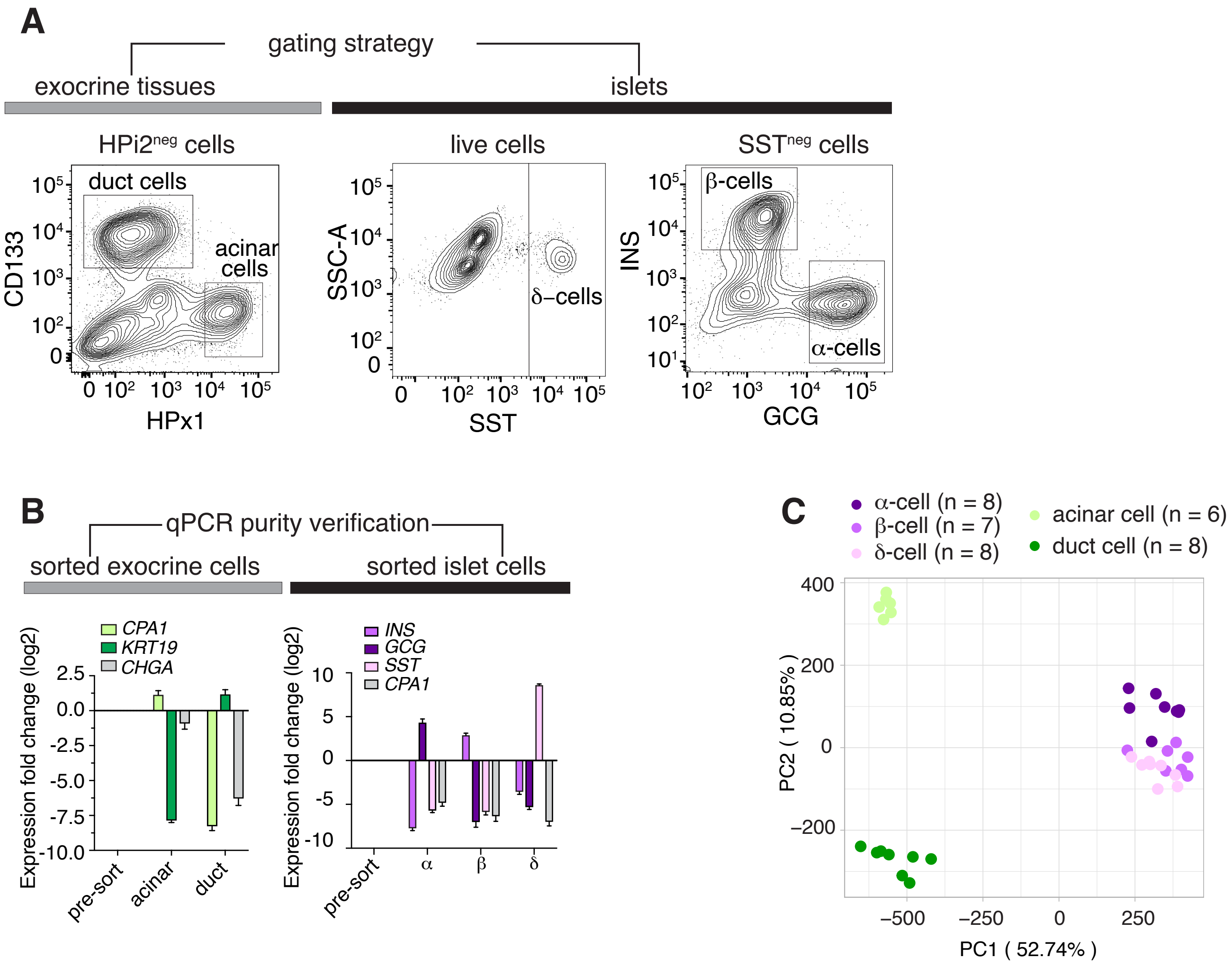

Supplementary Figure 1 (relates to Figure 1):

A. FACS plots showing the gating strategy to isolate different pancreas cell populations. Also see Supplementary Table 2.

B. Bar plots showing the enrichment and depletion of marker genes in each purified cell population as determined by qPCR analysis. Results were normalized to pre-sorted cells.

C. The Principal Component Analysis shows the clustering of ATAC-seq samples based on chromatin accessibility profiles across different pancreatic cell types. Each point represents a sample, and the samples are color-coded according to their respective cell type. The number of donors (n) used for each cell type is indicated.

bioRxiv preprint doi: https://doi.org/10.1101/2024.09.07.611794; this version posted September 13, 2024. The copyright holder for this preprint (which was not certified by peer review) is the author/funder. This article is a US Government work. It is not subject to copyright under 17 USC 105 and is also made available for use under a CC0 license.

Wang et al, Supplementary Figure 2

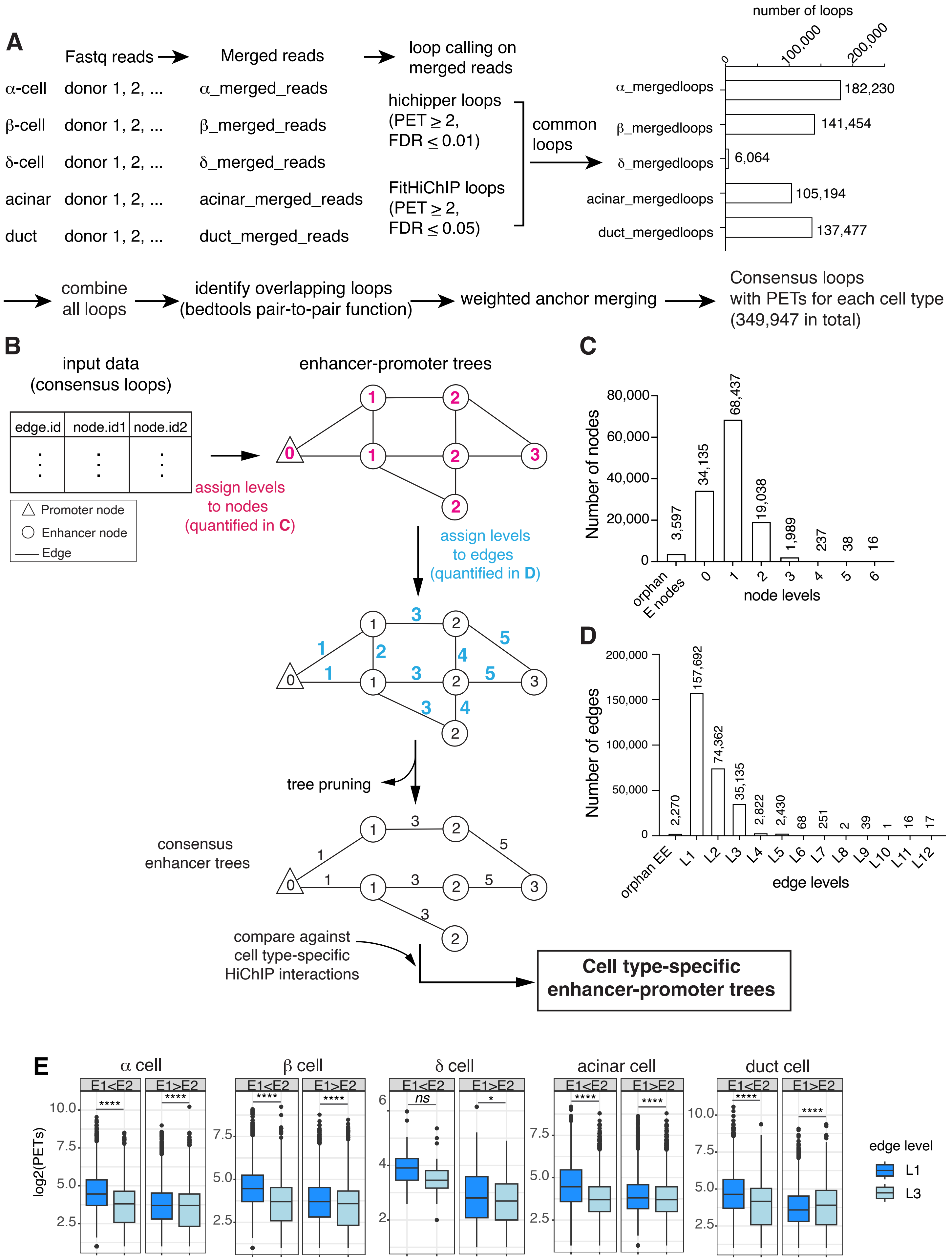

bioRxiv preprint doi: https://doi.org/10.1101/2024.09.07.611794; this version posted September 13, 2024. The copyright holder for this preprint (which was not certified by peer review) is the author/funder. This article is a US Government work. It is not subject to copyright under 17 USC 105 and is also made available for use under a CC0 license.

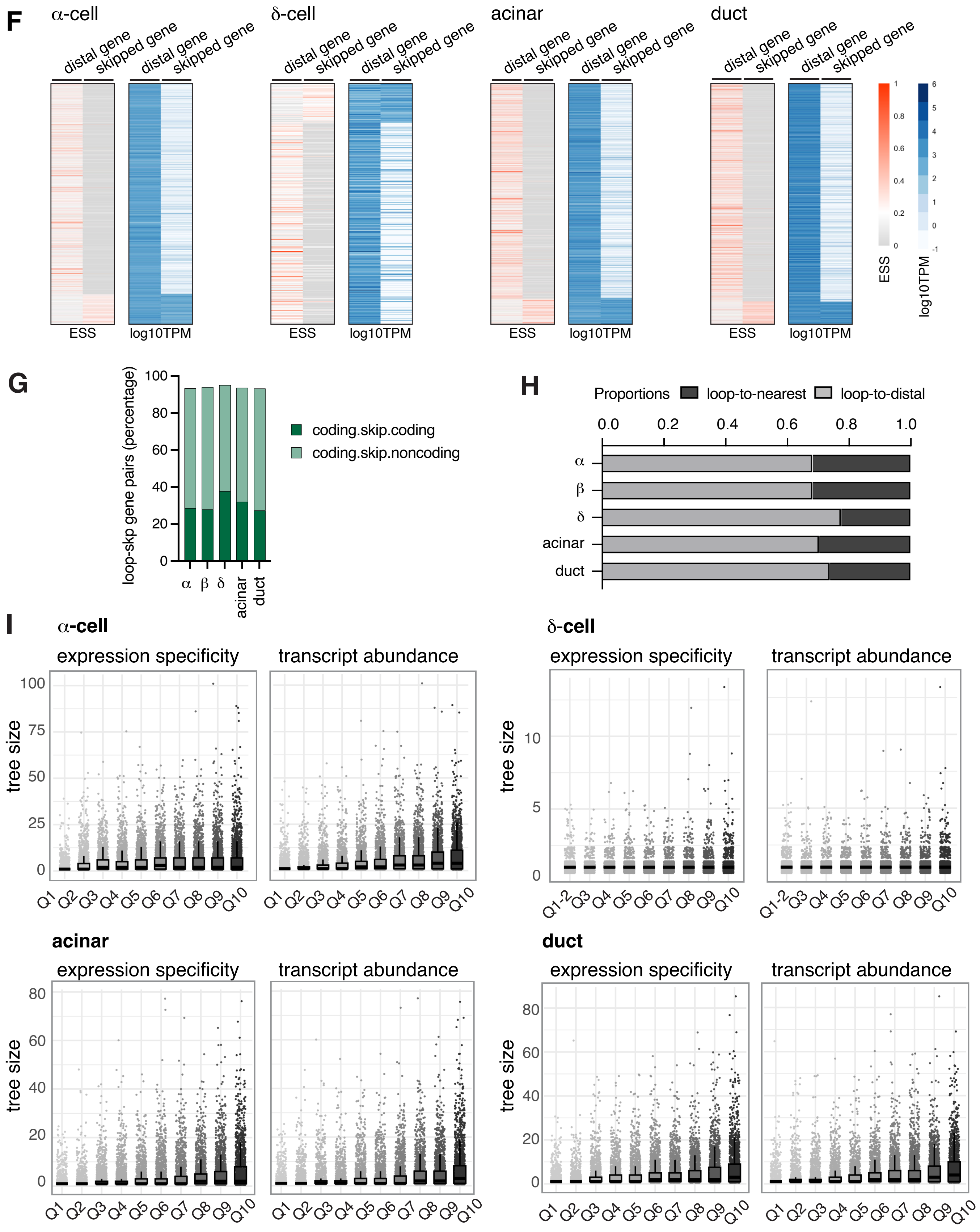

bioRxiv preprint doi: https://doi.org/10.1101/2024.09.07.611794; this version posted September 13, 2024. The copyright holder for this preprint (which was not certified by peer review) is the author/funder. This article is a US Government work. It is not subject to copyright under 17 USC 105 and is also made available for use under a CC0 license.

Supplementary Figure 2 (relates to Figure 2)

A. Flowchart describing consensus loop development.

B. Schematic detaling enhancer tree construction based on consensus loops.

C-D. Bar graphs depict the distribution of nodes (C) and edges (D) by connectivity level in enhancer trees before pruning.

E. Interaction frequency stratified by distance of E1 and E2 relative to their corresponding promoters. Mann-Whitney test, **** P-value < 0.0001; * P-value < 0.05; ns, not significant.

F. Heat maps show the expression specificity (ESS, red) and abundance (blue) of distally looped or skipped genes. Each row represents a gene pair that are either distally looped to or skipped by the same enhancer in α-, δ-, acinar or duct cells.

G. Distribution of gene pairs based on their type in distally looping and skipped gene interactions across different pancreatic cell types (α-, β-, δ-, acinar or duct cells.). The dark green bars represent the percentage of gene pairs where both the distally looping and skipped genes are coding genes (coding.skip.coding). The light green bars show the percentage of pairs where the distally looping gene is a coding gene, but the skipped gene is a non-coding gene (coding.skip.noncoding).

H. Fraction of enhancers looping to the nearest gene (dark grey) or a distal gene (light grey) in each cell type when noncoding genes are excluded from the datasets.

I. Box plots depict the relationship between transcript abundance and the size of enhancer-promoter trees as measured by the number of enhancers linked to a single promoter. The x-axis represents the quantiles of expression specificity (ESS) or transcript abundance. The individual data points represent specific tree sizes for genes within each quantile.

bioRxiv preprint doi: https://doi.org/10.1101/2024.09.07.611794; this version posted September 13, 2024. The copyright holder for this preprint (which was not certified by peer review) is the author/funder. This article is a US Government work. It is not subject to copyright under 17 USC 105 and is also made available for use under a CC0 license.

Wang et al, Supplementary Figure 3

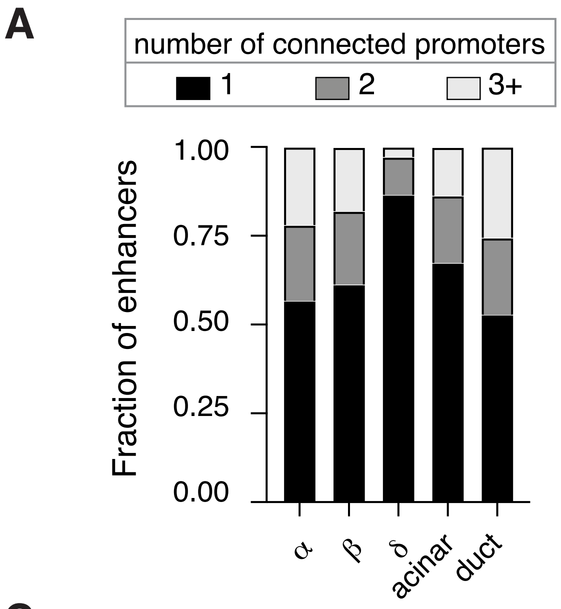

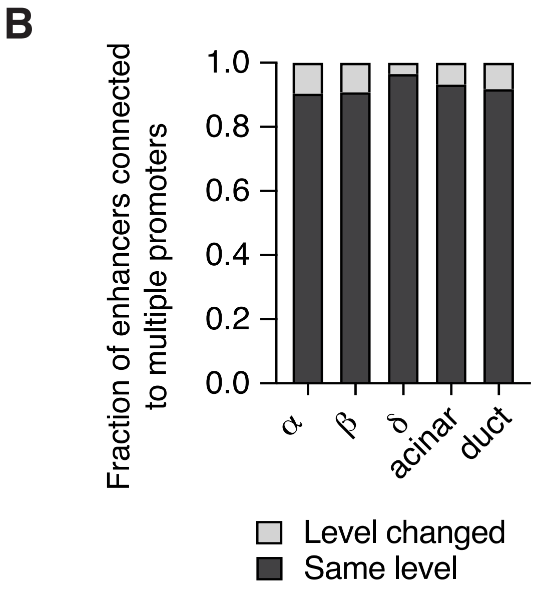

C

	α cell trees	β cell trees	acinar cell trees	duct cell trees
in forest	14813	13354	10094	12134
not in forest	744	922	855	349

Supplementary Figure 3 (relates to Figure 3)

A. Bar graph shows fraction of enhancers connecting to one, two, three or more promoters quantified in each pancreatic cell type.

B. Bar graph shows the proportion of enhancers that connect to multiple promoters in terms of level changes in each cell type.

C. Table showing the number of trees belonging to a forest, stratified by cell type.

bioRxiv preprint doi: https://doi.org/10.1101/2024.09.07.611794; this version posted September 13, 2024. The copyright holder for this preprint (which was not certified by peer review) is the author/funder. This article is a US Government work. It is not subject to copyright under 17 USC 105 and is also made available for use under a CC0 license.

Wang et al, Supplementary Figure 4

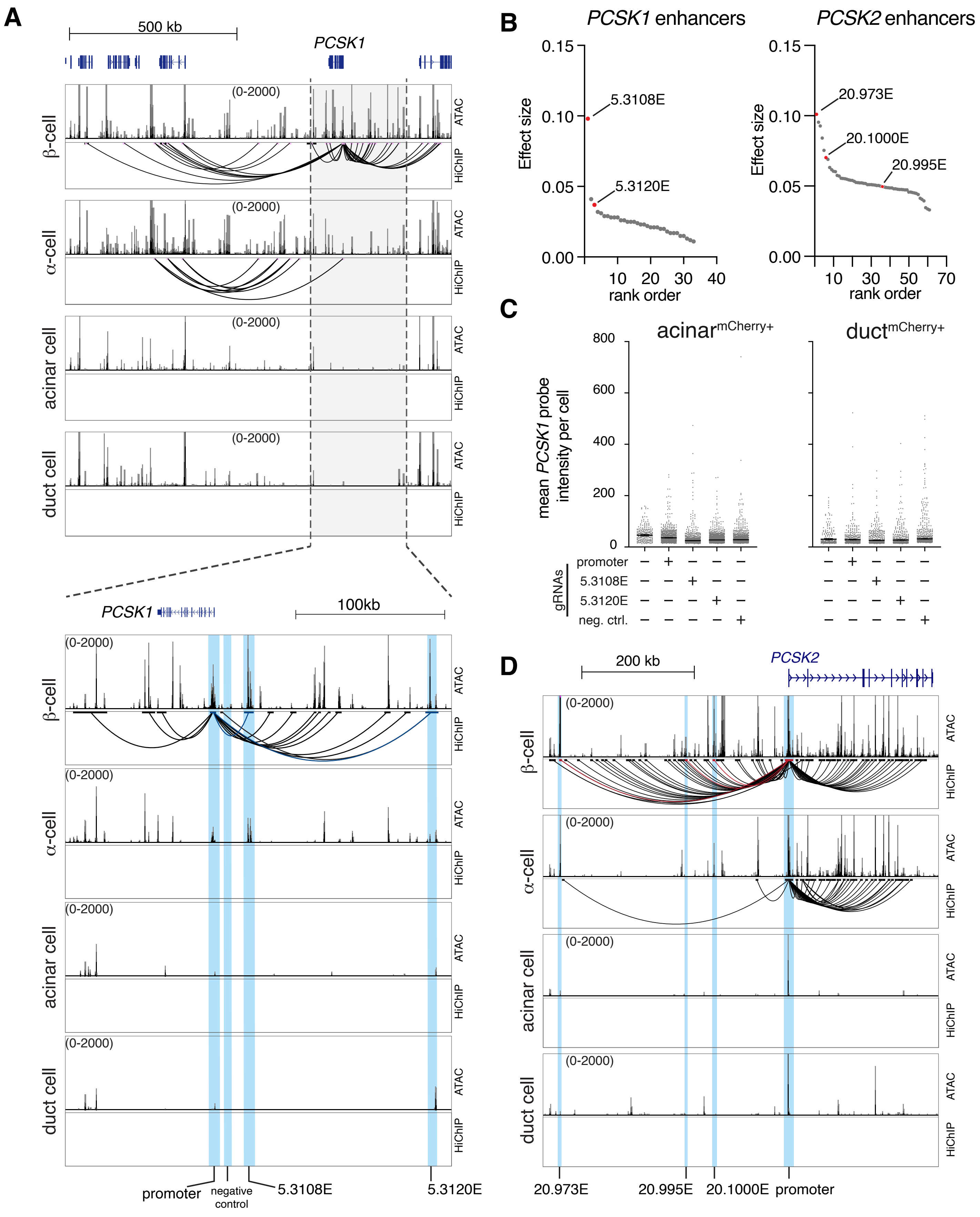

bioRxiv preprint doi: https://doi.org/10.1101/2024.09.07.611794; this version posted September 13, 2024. The copyright holder for this preprint (which was not certified by peer review) is the author/funder. This article is a US Government work. It is not subject to copyright under 17 USC 105 and is also made available for use under a CC0 license.

Supplementary Figure 4 (relates to Figure 5)

A. UCSC genome browser tracks displaying ATAC-seq peaks and HiChIP loops at the *PCSK1* locus across different pancreas cell types. The top set of tracks shows a broader region around the *PCSK1* gene, showing all the enhancer-promoter interactions corresponding to the *PCSK1* tree in β-cells. The bottom set of tracks zooms in on a region around *PCSK1,* blue highlighted regions indicate the top-ranking enhancers 5.3108E and 5.3120E, the promoter and the negative control regions.

B. Scatter plots displaying the ranked effect sizes of β-cell *PCSK1* tree enhancers (left) and α-cell *PCSK2* tree enhancers (right). Each point represents an enhancer, ordered by effect size. The enhancers that were tested in CRISPR perturbation assays are marked in red.

C. Quantification of *PCSK1* transcript levels in $mCherry^+$ acinar cells and $mCherry^+$ duct cells after CRISPRa targeting. Mean *PCSK1* probe intensity per cell is shown for cells treated with gRNAs targeting the promoter (positive control), enhancers 5.3108E and 5.3120E, and a negative control region. No activation was observed in either cell type or perturbation condition. n($mCherry^+$ acinar cells)= 4714, n($mCherry^+$ duct cells)= 2193, results were reproduced by at least two independent donors.

D. UCSC genome browser tracks displaying ATAC-seq peaks and HiChIP loops at the *PCSK2* locus across different pancreas cell types. Blue highlighted regions indicate the promoter and EPIC-prioritized enhancers regions.

bioRxiv preprint doi: https://doi.org/10.1101/2024.09.07.611794; this version posted September 13, 2024. The copyright holder for this preprint (which was not certified by peer review) is the author/funder. This article is a US Government work. It is not subject to copyright under 17 USC 105 and is also made available for use under a CC0 license.

Wang et al, Supplementary Figure 5

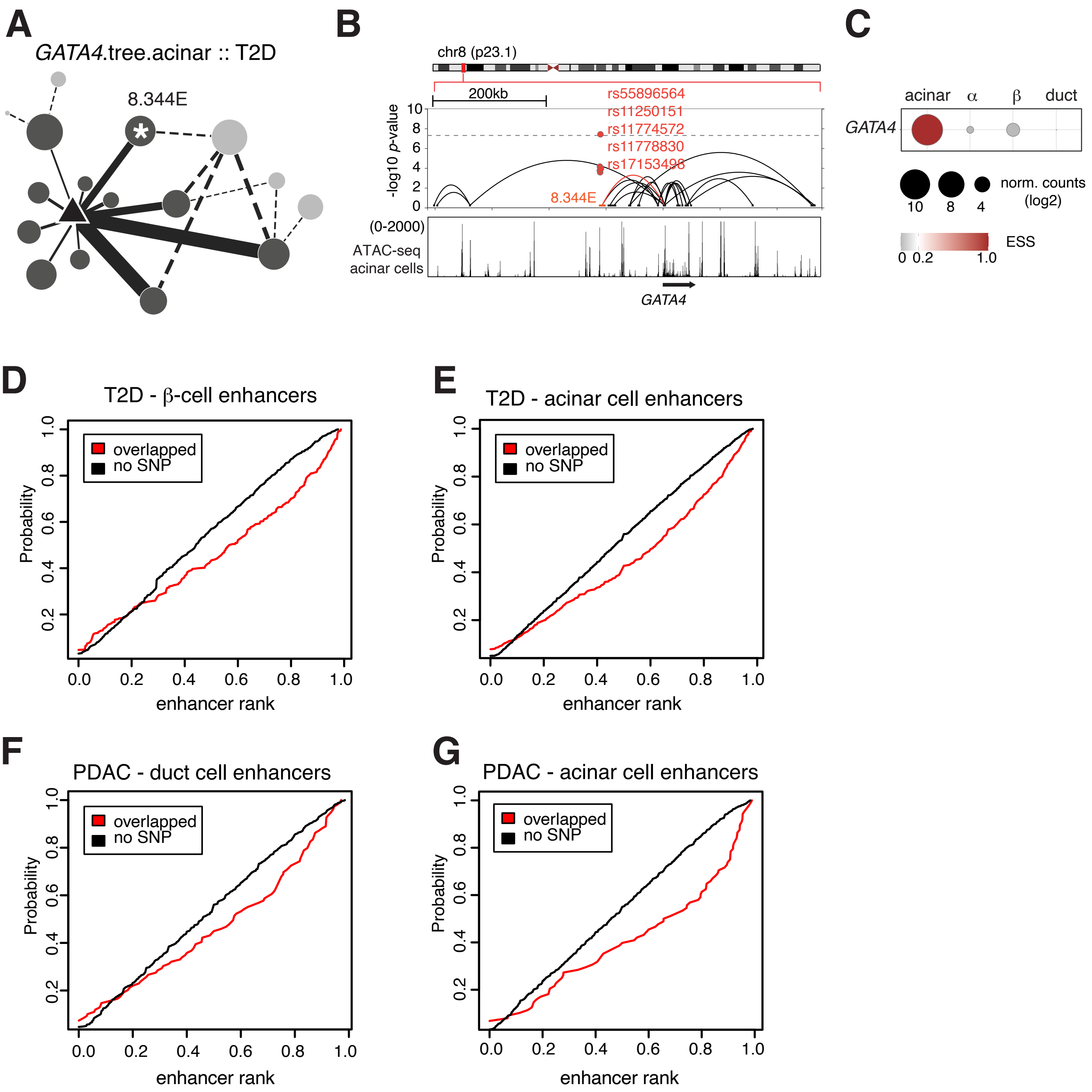

bioRxiv preprint doi: https://doi.org/10.1101/2024.09.07.611794; this version posted September 13, 2024. The copyright holder for this preprint (which was not certified by peer review) is the author/funder. This article is a US Government work. It is not subject to copyright under 17 USC 105 and is also made available for use under a CC0 license.

Supplementary Figure 5 (relates to Figure 6)

A. Network representation of the *GATA4* enhancer tree in acinar cells. Nodes represent enhancers, with the size of each node reflecting the ATAC-seq tag density and the thickness of the lines (edges) indicating the strength of the enhancer-promoter interactions as detected by HiChIP. The node with the asterisk indicates the SNP enriched enhancer.

B. Combined UCSC genome browser and locus zoom plots displaying the enhancer tree elements at the *GATA4* locus in acinar cells. The locus zoom plot highlighting significant SNPs in red. The UCSC genome browser tracks below show the corresponding ATAC-seq peaks and HiChIP loops detected in acinar cells. Red highlights the node enriched with the significant SNPs.

C. Bubble plot depicts the expression specificity (ESS) and transcript abundance (normalized counts) of *GATA4* transcripts in human pancreas cells based on single-cell RNA-seq data.

D-G. Cumulative Distribution Function (CDF) plots comparing the enhancer ranks based on EPIC's prioritization, with and without SNP overlap, for different cell types and GWAS traits. The top-ranking enhancer has the value of 1, and bottom-ranking enhancer has the value of zero.

(D) The red line represents enhancers that overlap with SNPs associated with type 2 diabetes (T2D) in β-cells, while the black line represents enhancers without SNP overlap.
(E) The same comparison for enhancers in acinar cells associated with T2D.
(F) Enhancers overlapping with SNPs (red) and those without SNP overlap (black) in ductal cells associated with pancreatic ductal adenocarcinoma (PDAC).
(G) The same comparison for enhancers in acinar cells associated with PDAC.
